# Evaluation of I-TAC as a potential early plasma marker to differentiate between critical and non-critical COVID-19

**DOI:** 10.15698/cst2022.01.262

**Published:** 2021-12-21

**Authors:** Yushan Zhang, Chao Xu, Nelson I. Agudelo Higuita, Resham Bhattacharya, Jennifer Holter Chakrabarty, Priyabrata Mukherjee

**Affiliations:** 1 Department of Pathology, University of Oklahoma Health Sciences Center, Oklahoma City, Oklahoma 73104, USA.; 2 Department of Biostatistics and Epidemiology, University of Oklahoma Health Sciences Center, Oklahoma City, Oklahoma 73104, USA.; 3 Department of Medicine, University of Oklahoma Health Sciences Center, Oklahoma City, Oklahoma 73104, USA.; 4 Department of Obstetrics and Gynecology, University of Oklahoma Health Sciences Center, Oklahoma City, Oklahoma 73104, USA.; 5 Peggy and Charles Stephenson Cancer Center, University of Oklahoma Health Sciences Center, Oklahoma City, Oklahoma 73104, USA.

**Keywords:** COVID-19, SARS-CoV-2, I-TAC, Gasdermin B, pyroptosis, disease severity

## Abstract

The COVID-19 pandemic has led to significant global health and economic consequences. There is an unmet need to define a molecular fingerprint of severity of the disease that may guide an early, rational and directed intervention preventing severe illness. We collected plasma from patients with moderate (nine cases), severe (22 cases) and critical (five cases) COVID-19 within three days of hospitalization (approximately one week after symptom onset) and used a cytokine antibody array to screen the 105 cytokines included in the array. We found that I-TAC, IP-10, ST2 and IL-1ra were significantly upregulated in patients with critical disease as compared to the non-critical (moderate and severe combined). ELISA further quantified I-TAC levels as 590.24±410.89, 645.35±517.59 and 1613.53±1010.59 pg/ml in moderate, severe and critical groups, respectively. Statistical analysis showed that I-TAC levels were significantly higher in patients with critical disease when compared with moderate (p = 0.04), severe (p = 0.03) or the combined non-critical (p = 0.02) group. Although limited by the low sample numbers, this study may suggest a role of I-TAC as a potential early marker to discriminate between critical and non-critical COVID-19 cases. Such knowledge is urgently needed for appropriate allocation of resources and to serve as a platform for future research towards early interventions that could mitigate disease severity and save lives.

## INTRODUCTION

The coronavirus disease 2019 (COVID-19), caused by the severe acute respiratory syndrome coronavirus 2 (SARS-CoV-2), is not a single entity but a disease with an extraordinary spectrum of phenotypes. The virus can cause asymptomatic and symptomatic infections, and when symptomatic, the severity can range from mild to critical. Mild and moderate disease require no oxygen supplementation in contrast to severe and critical disease. Severe illness is defined by a respiratory frequency greater than 30 breaths per minute, oxygen saturation of <94% on room air at sea level, ratio of arterial partial pressure of oxygen to fraction of inspired oxygen <300 mmHg, or lung infiltrates >50% [1, 2]. Individuals with critical stage require either ventilatory support, extracorporeal membrane oxygenation (ECMO), proning, and management of septic shock or organ dysfunction [3, 4]. The in-hospital mortality is approximately 15-20% but can be as high as 40% for those admitted to the intensive care unit (ICU) dependent on coexistent comorbidities (**Table 1**) [5, 6]. Particularly, almost all deaths happened in the critical stage [1].

Specific treatment interventions against COVID-19 are limited, imperfect, and are currently geared to those with severe and critical disease. Theoretically, antivirals and neutralizing antibodies (e.g. convalescent plasma, hyperimmune globulins or monoclonal antibodies) will be more effective when administered early in the course during active viral replication. However, the only antiviral in clinical use (i.e. remdesivir) is most effective in those with severe disease who do not require high-flow supplemental oxygen or ventilatory support. The medication does not alter outcome but does decrease the time of recovery [7]. A maladaptive host immune response plays an important role in the development of complications that tend to occur later in the disease course. The uncontrolled pro-inflammatory response leads to quantitative and qualitative lymphocyte, monocyte, granulocyte and platelet abnormalities (**Table 1**) [8]. Anti-inflammatory and immunomodulatory agents may be useful in this stage. For example, several agents have been examined in clinical trials, with only dexamethasone and interleukin-6 receptor antagonists improving outcome. Notably, dexamethasone only was beneficial in patients that have been symptomatic for at least seven days and who required oxygen [9] and tocilizumab and sarilumab have been shown to improve outcome, including survival [10]. Whether the administration of other immunomodulators earlier in the disease course can prevent or ameliorate the pathological immune response is unknown.

Although there are several clinical risk factors, laboratory abnormalities, and immune patterns [8, 11–13] that have been associated with an increased risk for poor outcome, tools to accurately predict the natural course of the disease soon after infection are currently not available. The response to existing treatment modalities is not uniform and is determined by viral, host and environmental factors. There is therefore an urgent and unmet need to identify a molecular fingerprint of severity that will guide early, rational, and directed interventions that could potentially influence outcome. An accurate and predictive diagnostic tool to predict severity would be useful both for identification of at-risk populations and early intervention with therapeutics that will mitigate severity and save lives. In this study, we checked the cytokine level changes in plasma of 36 COVID-19 patients, and evaluated I-TAC as a potential early plasma marker to predict disease progression.

## RESULTS AND DISCUSSION

### Patient demographics, clinical characteristics and sample collection

Plasma from 36 adult patients enrolled in the COVID-19 biorepository at the University of Oklahoma IRB #11911 and three SARS-CoV-2 negative control plasma samples were evaluated. The patients were stratified as at mild, moderate, severe or critical stage based on the level of respiratory support required and need for admission (no O_2_ requirement, O_2_ requirement up to 6 L, high flow or bipap required, ventilation required). Only moderate, severe or critical patients were hospitalized. The vast majority of patients had been admitted around one week (at the end of the week or beginning of the second week) after symptom onset. Diagnosis was confirmed by nasopharyngeal PCR assay at the day or the next day of admission. The final stage of the disease each patient developed was determined during the disease course. Most of the baseline plasma samples were drawn within three days of hospitalization and before administration of any immunomodulatory agents. These samples represented nine moderate, 22 severe and five critical diseases. Clinical characteristics including age, sex, lymphocyte count, neutrophil count, ferritin, liver function tests, creatinine, hemoglobin, INR (prothrombin time international normalized ratio), comorbidities, concomitant medications, and hospitalization time were collected. Status at discharge were recorded (**Table 1**).

**TABLE 1.** Patient demographics and clinical characteristics.

	**Moderate**	**Severe**	**Critical**	**All**
**Demographics**				
Number, N	9	22	5	36
Age, Median (Range)	43 (23-67)	60 (29-80)	53 (39-61)	54 (23-80)
Sex, Male	4 (44.4%)	11 (50%)	2 (40%)	17 (47.2%)
Female	5 (55.6%)	11 (50%)	3 (60%)	19 (52.8%)
Race				
African American	2 (22.2%)	4 (18.2%)	0	6 (16.7%)
American Indian or Alaska Native	0	1 (4.5%)	0	1 (2.8%)
White	5 (55.6%)	14 (63.6%)	4 (80%)	23 (63.9%)
Other	1 (11.1%)	2 (9.1%)	1 (20%)	4 (11.1%)
Unknown (Not Reported)	1 (11.1%)	1 (4.5%)	0	2 (5.6%)
**Comorbidities**				
Hypertension	5 (55.6%)	12 (54.5%)	2 (40%)	19 (52.8%)
Diabetes	3 (33.3%)	9 (40.9%)	3 (60%)	15 (41.7%)
Hypothyroidism	2 (22.2%)	4 (18.2%)	1 (20%)	7 (19.4%)
Asthma	0	3 (13.6%)	0	3 (8.3%)
Cancer	0	3 (13.6%)	0	3 (8.3%)
Coronary Artery Disease	2 (22.2%)	1 (4.5%)	0	3 (8.3%)
Obesity	0	2 (9.1%)	1 (20%)	3 (8.3%)
Anxiety	0	1 (4.5%)	1 (20%)	2 (5.6%)
Obstructive Sleep Apnea	0	0	2 (40%)	2 (5.6%)
**Admission Lab Tests** Median (Range)				
Neutrophil [2.2-7.8 × 10^9^/L]	5.36 (3-9.09)	6.52 (3.31-10.79)	3.13 (2.38-7.28)	5.36 (2.38-10.79)
Lymphocyte [0.9 – 3.3 × 10^9^/L]	0.83 (0.5-1.85)	0.775 (0.24-2.53)	0.8 (0.6-0.99)	0.79 (0.24-2.53)
Hemoglobin [11.9-17.7 g/dL]	13.1 (8.6-13.8)	13.4 (8.5-16.6)	12.2 (11.4-15.4)	13.1 (8.5-16.6)
Platelets [150-350 × 10^9^/L]	209 (146-327)	246 (145-302)	173 (142-257)	211 (142-327)
C-reactive protein (CRP) [0.0-8.0 mg/L]	41 (4.5-227.1)	133.4 (14-301.6)	141.6 (76.6-244.7)	120.8 (4.5-301.6)
Ferritin [15-200 µg/L]	133.45 (7.5-303.9)	634.65 (34.4-8087.6)	681 (293.1-1548.2)	420.6 (7.5-8087.6)
Alanine transaminase (ALT) [0-35 units/L]	59 (20-389)	69 (34-95)	85 (25-169)	69 (20-389)
Aspartate transaminase (AST) [0-35 units/L]	80 (23-408)	50 (29-126)	138 (30-423)	80 (23-423)
Alkaline phosphatase (ALP) [36-92 units/L]	106 (68-216)	91 (57-174)	57 (37-105)	91 (37-216)
Total Bilirubin [0.3-1.2 mg/dL]	1.6 (0.2-2)	1.2 (0.4-1.5)	0.4 (0.3-0.7)	0.9 (0.2-2)
Creatinine [7-13 mg/L]	0.85 (0.76-1.17)	1.08 (0.7-1.57)	0.94 (0.72-1.01)	0.94 (0.7-1.57)
INR [0.8-1.1]	1.1 (1-1.2)	1.2 (1-1.3)	1.2 (1.2-1.6)	1.2 (1-1.6)
**Concomitant Medications**				
Rocephin	6 (66.7%)	15 (68.2%)	4 (80%)	25 (69.4%)
Dexamethasone	3 (33.3%)	17 (77.3%)	3 (60%)	23 (63.9%)
Azithromycin	4 (44.4%)	13 (59.1%)	4 (80%)	21 (58.3%)
Remdesivir	0	14 (63.6%)	5 (100%)	19 (52.8%)
Convalescent Plasma	0	10 (45.5%)	5 (100%)	15 (41.7%)
Hydroxychloroquine	1 (11.1%)	2 (9.1%)	2 (40%)	5 (13.9%)
Tocilizumab	0	1 (4.5%)	3 (60%)	4 (11.1%)
**Inpatient Status**				
Supplemental Oxygen	0	22 (100%)	5 (100%)	27 (75%)
>6L O2	0	11 (50%)	5 (100%)	16 (44.4%)
Ventilation	0	0	5 (100%)	5 (13.9%)
ECMO	0	0	1 (20%)	1 (2.8%)
**Hospitalization Days** Median (Range)	2 (2-9)	10 (1-26)	21 (16-34)	9 (1-34)
**Discharge Status**				
Home	8 (88.9%)	20 (90.9%)	1 (20%)	29 (80.6%)
Nursing Home	1 (11.1%)	0	0	1 (2.8%)
Skilled Nursing Facility	0	0	1 (20%)	1 (2.8%)
Rehab Hospital	0	0	1 (20%)	1 (2.8%)
Long-Term Acute Care facility	0	1 (4.5%)	0	1 (2.8%)
Hospice	0	1 (4.5%)	0	1 (2.8%)
Death	0	0	2 (40%)	2 (5.6%)

### Screening of cytokines associated with COVID-19 severity

**Figure 1:**
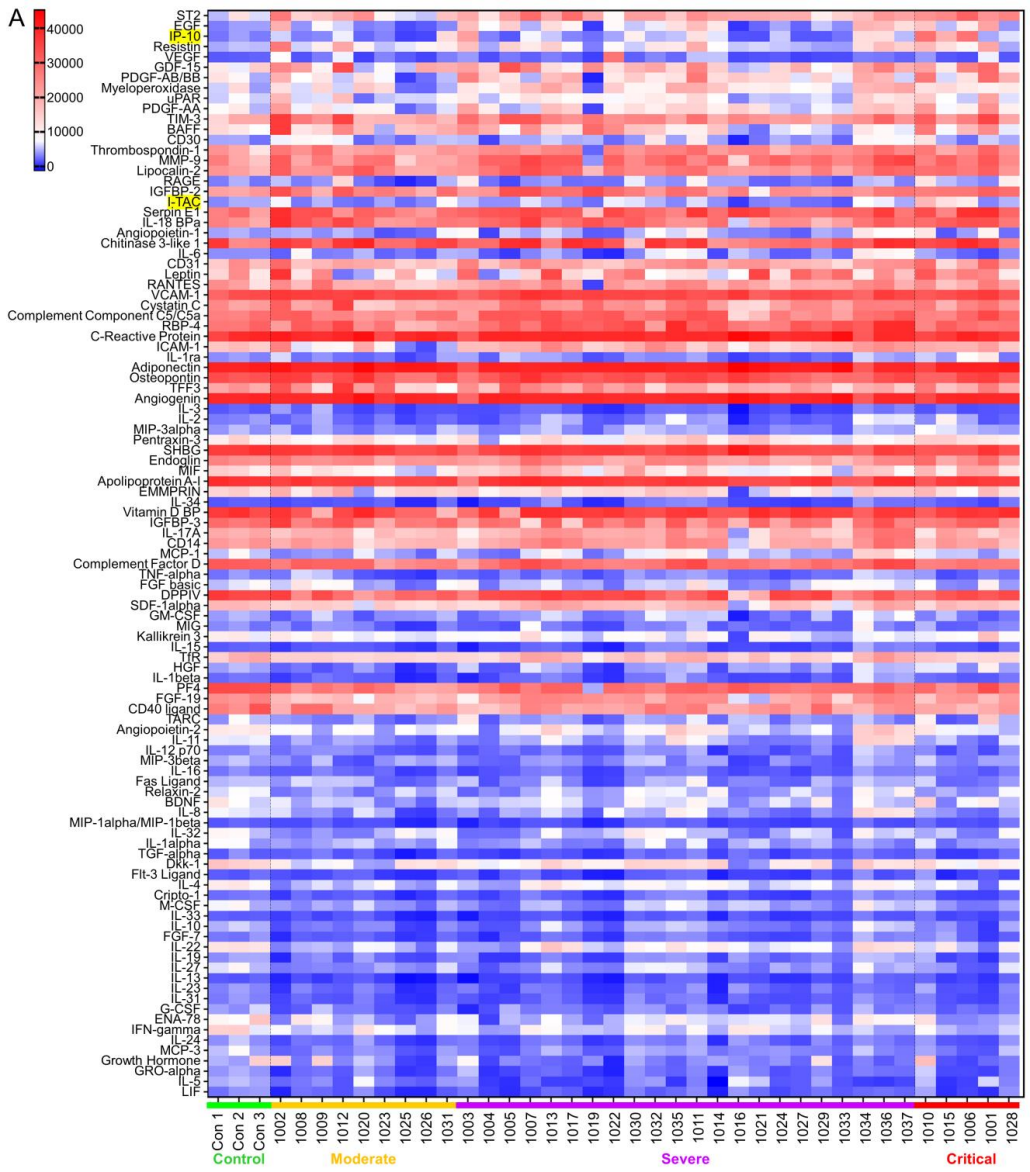

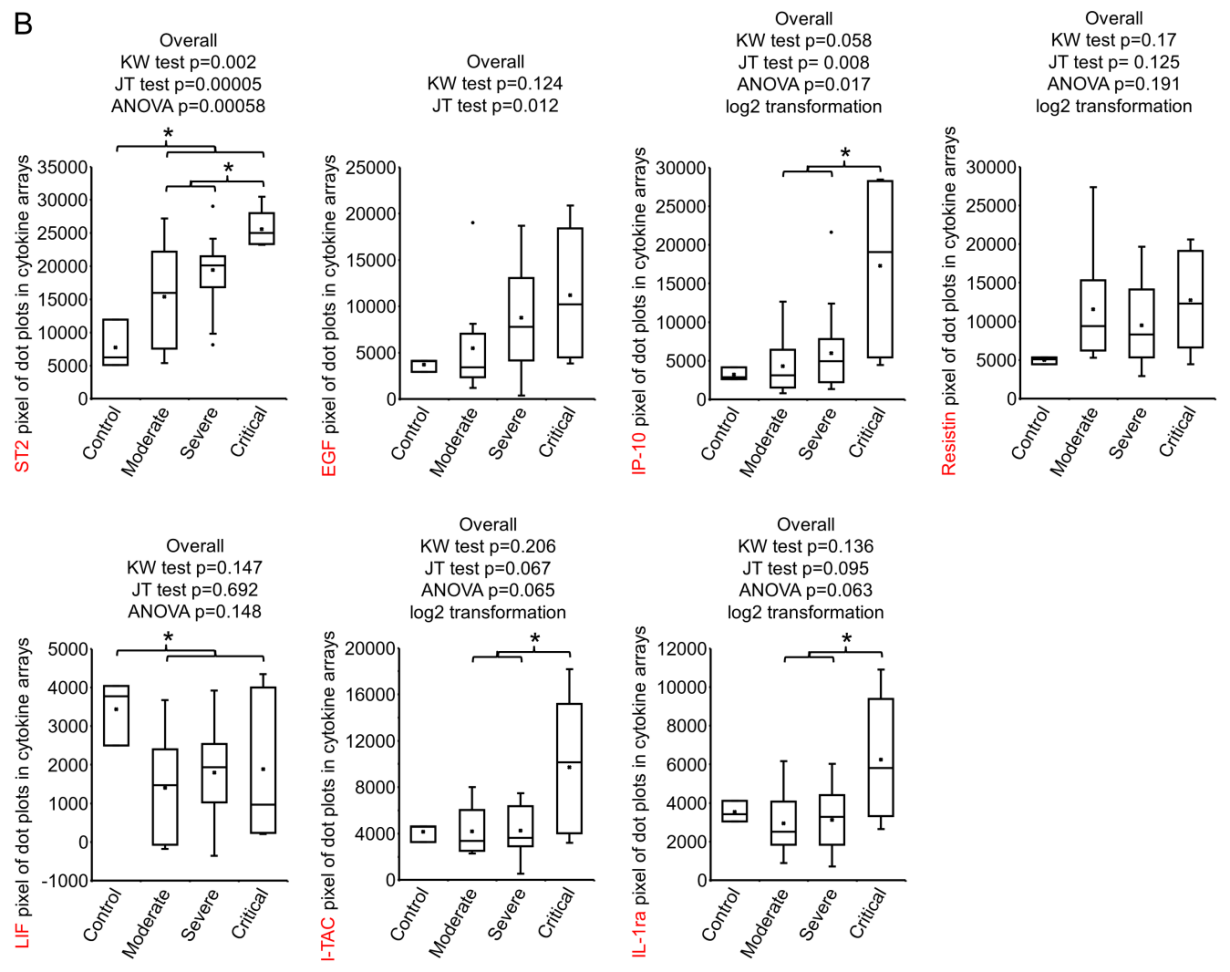
FIGURE 1: Screening of early plasma cytokines. **(A)** Human XL Cytokine Array Kits were used for cytokine screening. 20 μl plasma from each patient were diluted with 1.5 ml Array Buffer 6 and applied to one Cytokine Antibody Array Membrane. Blot images were quantified with Quick Spot image analysis software and normalization among batches of assays was done against positive controls. Shown is the expression heatmap of the cytokines. Cytokines were arranged according to the expression ratio of disease (moderate + severe + critical, n= 9 + 22 + 5 =36) to normal control (n=3). Numbers 1001-1017, 1019-1037 on the bottom: COVID-19 patient ID; Con: normal control. I-TAC and IP-10 on the left column are highlighted. **(B)** Cytokines whose expression ratio between disease and control or between critical and non-critical was higher than 2 or lower than 0.5 was subjected to statistical analysis. Raw values were tested for normality distribution. ST2 and LIF passed the test and ANOVA test were performed. Log2 transformation was made to the other five cytokines. IP-10, Resistin, I-TAC and IL-1ra passed the normality test and ANOVA test were performed using transformed values. EGF, as well as all other cytokines here, was subjected to non-parametric tests Kruskal-Wallis rank sum (KW) test to test overall difference among four groups and Jonckheere-Terpstra k-sample (JT) test to test if there is increasing or decreasing trend among the groups. *, p<0.05 with ANOVA test. The pairwise expression comparisons between disease and control or between critical and non-critical for EGF was done with Wilcoxon rank sum test and no significance reached

SARS-CoV-2 infection causes the host cells to undergo pyroptosis, a highly inflammatory form of programmed cell death [14, 15], releasing IL-1β, pathogen-associated molecular patterns (PAMPs) and damage-associated molecular patterns (DAMPs). The neighboring cells recognize the molecules and patterns and, at a very early stage of disease, secrete pro-inflammatory cytokines including IL-6, IFN, IP-10, MIP-1 and MCP-1 which further trigger a cytokine storm [14, 16, 17]. Despite reports that elevated levels of a few cytokines can be detected five to ten days from symptom onset in patients with severe or critical disease, predictive associations with disease severity have not been established [18]. We posited that some cytokines would be among the earliest molecules whose plasma levels may change upon symptom onset and therefore could be used as differentiator for disease severity. Using the Human XL Cytokine Array, we determined expression levels of 105 cytokines in the 36 plasma samples collected within three days of hospitalization (refered to as early plasma in the following description) as well as three normal controls. A typical image of the array for each sample is shown in **Fig S1**. After quantification of the original images using Quick Spot analysis software, and normalization among batches of assays against the positive spots in each array, an expression heatmap of the cytokines was generated, by ranking the fold changes between disease group (all COVID-19 cases together) vs control group (highest top, **Fig. 1A**). ST2, EGF, IP-10 and Resistin were upregulated 2.49, 2.22, 2.21 and 2.10 folds, respectively, whereas LIF was downregulated 0.5 folds in disease group vs control group. Comparison of the critical group with the non-critical groups put together (moderate + severe) showed that IP-10, I-TAC and IL-1ra levels were increased by 3.14, 2.29 and 2.02 folds, respectively (**Fig. 1B**). Statistical analysis of these seven cytokines showed that there were overall significant differences (p<0.05) among the four groups (control, moderate, severe and critical) for ST2, EGF, IP-10, LIF, I-TAC and IL-1ra. Specifically, ST2 and LIF showed significant differences between control and disease, whereas ST2, IP-10, I-TAC and IL-1ra showed significant differences between critical and non-critical (**Fig. 1B**). Both ST2 and IL-1ra are members of the IL-1 superfamily and IL-1 receptor blockade has improved clinical outcomes in cohort studies of COVID-19 [19], although efficacy has not been tested in controlled settings. We proceeded to quantify levels of IP-10 and I-TAC in the plasma from the same patients by ELISA.

### IP-10 level in COVID-19 early plasma

**Figure 2:**
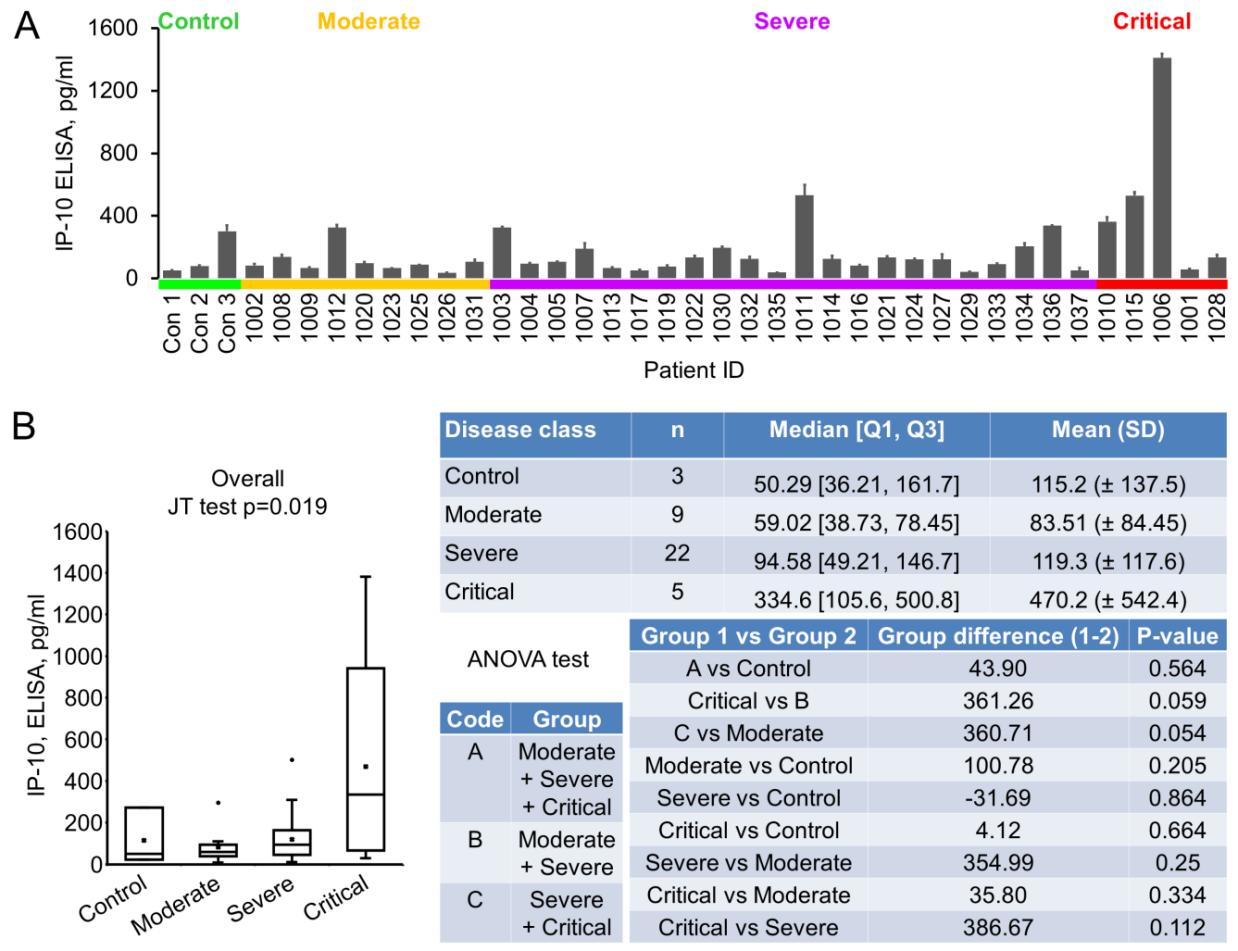
FIGURE 2: IP-10 levels in early plasma samples. **(A)** IP-10 levels were measured using ELISA kits. Plasma (10 µl) were diluted to 100 µl and added to anti-IP-10 antibody coated wells in duplicate. Data were groups by disease stage and expressed as mean ± SD. **(B)** Statistical analysis of IP-10 levels with disease stages.

IP-10 (Interferon gamma-induced protein 10), also known as CXCL10 (C-X-C motif chemokine ligand 10), a 98 amino acids cytokine of the CXC chemokine family, was reported to be upregulated in COVID-19 [17, 20]. Its early quantification, in combination with IL-6 and IL-10, was recently demonstrated to correlate with disease progression and length of hospitalization [21]. Our ELISA results showed that the average level of IP-10 in control, moderate, severe and critical groups were 115.20±137.52, 83.51±84.45, 119.31±117.58 and 470.18±542.44 pg/ml, respectively (**Fig. 2A, 2B**). Even though an overall significant difference was seen, indicating that there was an increasing trend (in the order of normal control, moderate, severe and critical) of this cytokine, no significance was reached between control and disease, or between critical and non-critical groups (**Fig. 2B**).

### I-TAC was quantified to differentiate critical disease from non-critical

**Figure 3:**
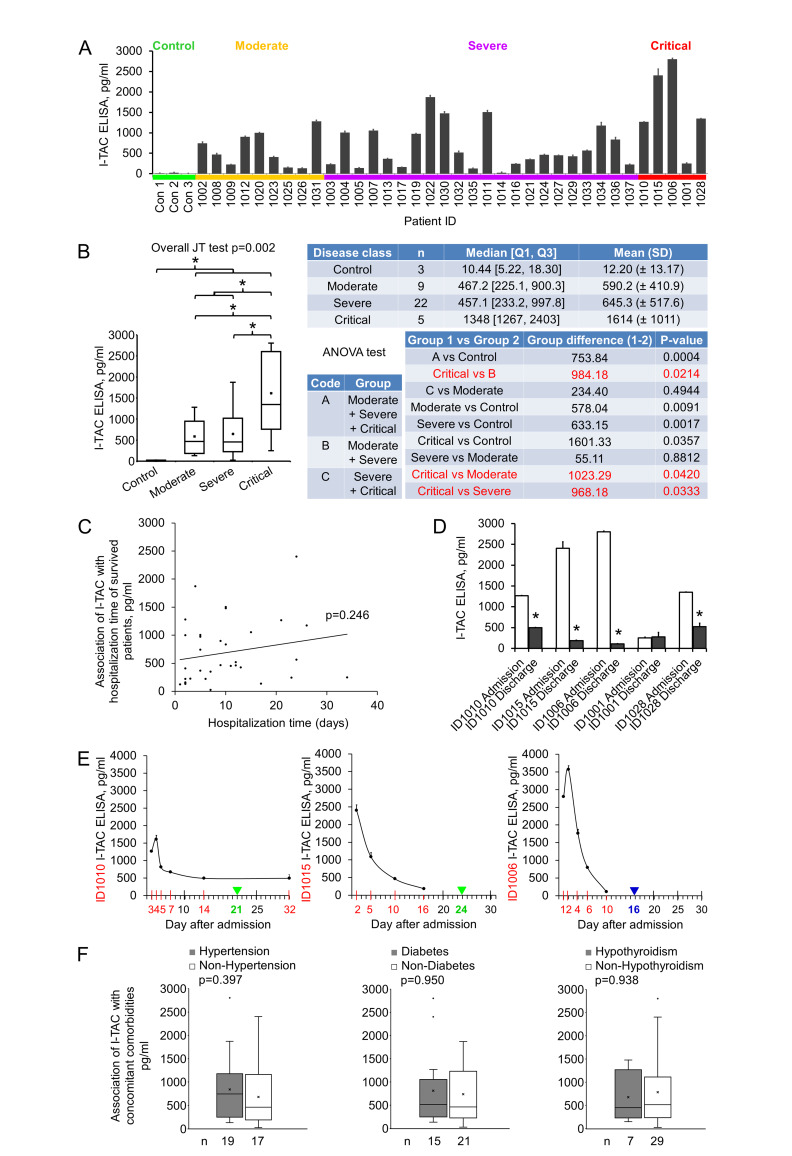
FIGURE 3: I-TAC levels in patient plasma. **(A)** I-TAC levels were measured using ELISA kits. Early plasma (25 µl) were diluted to 100 µl and added to anti-I-TAC mixture coated wells in duplicate. Data were groups by disease stage and expressed as mean ± SD. **(B)** Statistical analysis of I-TAC levels with disease stages. **(C)** Association of early plasma I-TAC levels with hospitalization time of survived patients (n=34). **(D)** I-TAC levels at time closest to discharge. ID1010: 7 days before discharge; ID1015: 8 days before discharge; ID1006: 6 days before discharge; ID1001: 2 days before discharge; ID1028: 7 days before discharge. Admission: within 3 days of hospitalization. **(E)** Changes of I-TAC level after admission. Red number day: sample collection time. Green number day and green triangle: discharged live. Blue number day and blue triangle: discharged deceased. **(F)** Association of early plasma I-TAC with concomitant comorbidities. Comorbidities and patient numbers in each group (n) were indicated. *, p<0.05.

I-TAC (Interferon-inducible T-cell alpha chemoattractant), also known as CXCL11, is a 94 amino acids cytokine of the CXC chemokine family. I-TAC is highly expressed in peripheral blood leukocytes, pancreas and liver, moderately or lowly expressed in thymus, spleen, lung, placenta, prostate, and small intestine [22]. Upon the induction of its expression by interferon at sites of inflammation, I-TAC primarily functions as an inflammatory chemotactic factor by binding to its receptor CXCR3 and CXCR7 on immune cells such as activated T cells and attracting these cells to the site of injury [23, 24]. I-TAC was shown to be upregulated at the early stage (eight to nine mean days after disease onset) of COVID-19 in ICU, non-ICU, and the mild disease groups, but no significant differences were detected among the groups [20]. We measured early plasma I-TAC levels by ELISA. I-TAC in control, moderate, severe and critical groups were 12.20±13.17, 590.24±410.89, 645.35±517.59 and 1613.53±1010.59 pg/ml, respectively (**Fig. 3A, 3B**). We found that I-TAC levels were significantly increased in the COVID-19 disease group (all stages combined) compared with the normal control group (p = 0.0004) (**Fig. 3B**). Importantly, I-TAC levels were significantly increased in critical patients when compared with moderate (p = 0.04), severe (p = 0.03) or the combined non-critical (moderate + severe) group (p = 0.02; **Fig. 3B**).

No significant difference between moderate and severe groups was detected. Of note, no significant association (p > 0.05) between I-TAC levels and hospitalization time of the surviving patients (**Fig. 3C**) was observed. We checked I-TAC in plasma collected at dates closest to discharge (two to eight days before discharge) for critical patients, and found that four patients experienced a dramatic decrease in I-TAC level, and one remained at a low level (**Fig. 3D, Table 1** for hospitalization time). For the critical patients whose plasma were available at more than two time points, I-TAC levels demonstrated a high-(higher)-low-lower curve with disease course (**Fig. 3E**). To examine whether there were some possible covariates or confounders that might influence early plasma I-TAC levels, we analyzed the association of the most common concomitant comorbidities (hypertension, diabetes and hypothyroidism, n≥7) with I-TAC (**Fig. 3F, Table 1** for comorbidities). No significant association (p > 0.05) was found. These results suggest that I-TAC may be a potential early plasma maker to differentiate between critical and non-critical COVID-19 patients, and may be worth further investigation.

### GSDMB is associated with COVID-19

**Figure 4:**
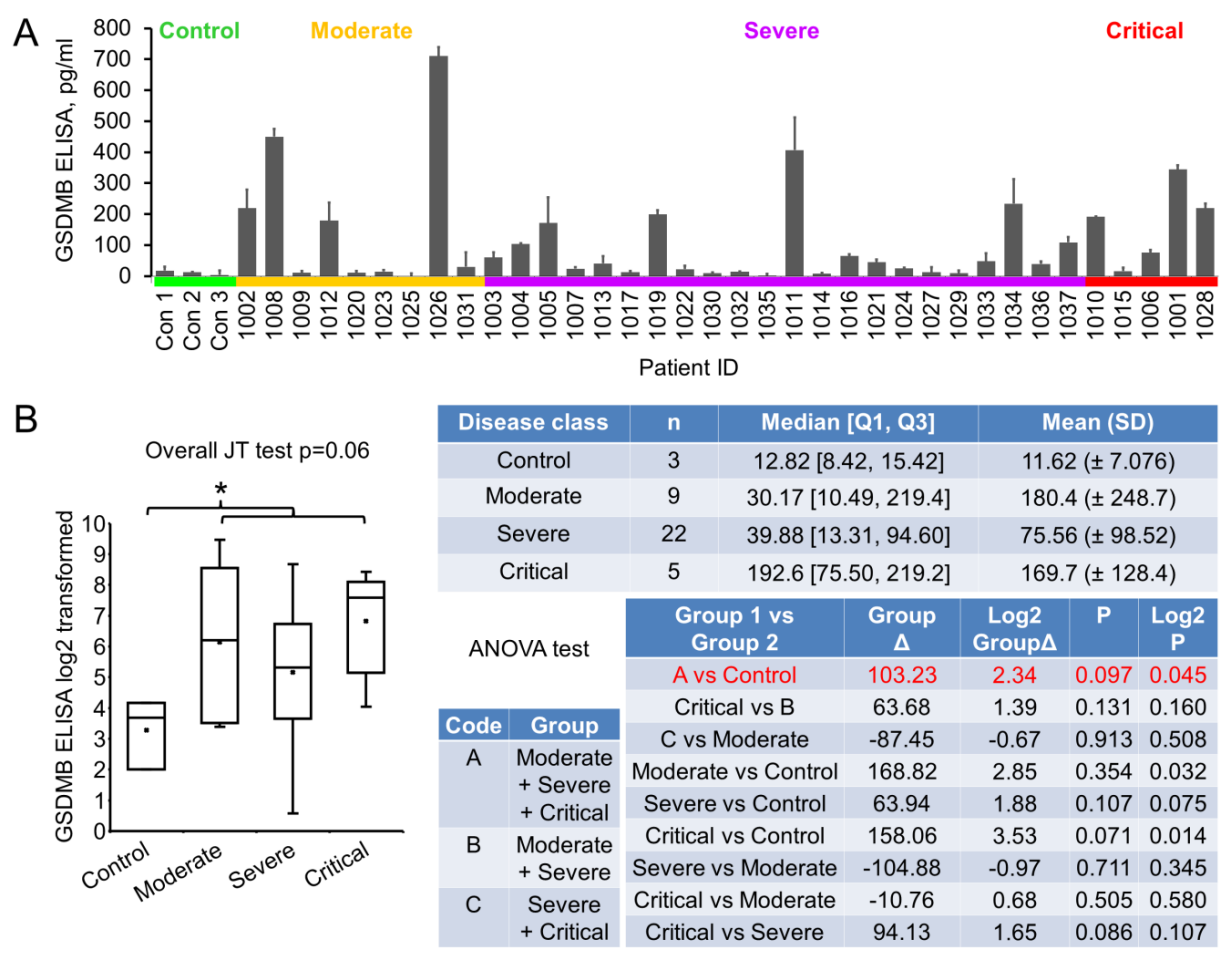
FIGURE 4: Gesdermin B expression in early plasma. **(A)** Plasma GSDMB levels were determined with ELISA. Plasma (50 µl) were diluted to 100 µl and added to anti-GSDMB coated wells in duplicate. Data were groups by disease stage and expressed as mean ± SD. **(B)** Statistical analysis of GSDMB levels with disease stages. Graph shows log2 transformation of the original values. Note that one original value in moderate group was 0, so the transformed meaningless value was removed, resulting in the decreased average compared to that in critical group. *, p<0.05 with ANOVA test. Δ, difference.

The severity of COVID-19 is known to associate with patient inflammatory response, including pyroptosis, of which Gasdermin B (GSDMB) is an important mediator [14, 15]. GSDMB is a member of the Gasdermin protein family that regulates pyroptotic inflammation and homeostasis [15, 25]. GSDMB expression was detected to be upregulated in human bronchial epithelial cells from asthmatics and related to the severity and exacerbation of asthma [26, 27]. We tried to explore any association of GSDMB with COVID-19 severity during the investigation. Pioneer immunoblotting suggested that GSDMB expression was elevated in the majority of the early plasma samples. ELISA was performed and significant upregulation of GSDMB in patient plasma was observed (9.89 folds increase, p=0.045): while in the normal plasma the GSDMB level was 11.62±7.08 pg/ml, it was 114.85±156.06 pg/ml in patient plasma (**Fig. 4**). However, with levels in moderate, severe and critical groups being 180.4±248.7, 75.56±98.52 and 169.7±128.4 pg/ml, respectively, no statistically significant association of GSDMB levels with disease severity could be established. Of note, GSDMB level in patients with asthma (three patients with severe COVID-19, **Table 1**) was not statistically different from that in controls, that in all other COVID-19 patients, or that in other severe COVID-19 patients, suggesting that COVID-19 may have an impact comparable to or even bigger than asthma on plasma GSDMB level change. These results suggest that GSDMB may be an early indicator for COVID-19, but not one predicting the prognosis of the disease.

In this study, we demonstrated that I-TAC and GSDMB are associated with COVID-19, and that I-TAC expression in early-disease plasma samples may differentiate between patients that will develop critical versus non-critical disease. It is important to note that using the cytokine array we have also detected elevated expression of ST2 and IL-1ra (members of the IL-1 superfamily) in critical disease. Consistent with this result, Patel *et al.* and Zeng *et al.* reported that ST2 or IL-1ra was associated with disease severity [28, 29]; Cavalli *et al.* observed that IL-1 receptor blockade improved clinical outcomes in cohort studies of COVID-19 [19]. These results support our methodologies to identify key molecules that can differentiate severity of COVID-19.

Haljasmagi *et al.* showed that I-TAC levels at the early stage of COVID-19 tended to increase with disease severity (from mild, non-ICU, to ICU), even though there were no significant differences among the groups [20]. We showed here that I-TAC levels at the early stage were significantly increased in critical compared to non-critical disease. Both studies supported the trend that I-TAC is upregulated with disease severity. The discrepancy in statistical significance between the two studies may come from the classification of severity. Haljasmagi *et al.* classified the moderate disease as non-ICU, and the severe (and critical) diseases as ICU. We classified moderate and severe as non-critical, and compared that with critical. Another possible reason could be that the sampling time was not exactly the same in the two studies, and, noteworthy, I-TAC levels change dramatically with sampling time (**Fig. 3E**). Additionally, the two studies employed different techniques, i.e., Proximity Extension Assay (target protein initiated real-time PCR) vs Antibody Array Assay + ELISA.

### Limitations of the study

As common for hypothesis-generating studies, there are limitations in this study as well. First, the available sample numbers were small, especially for the critical cases. This leads to wide confidence intervals (CI) of the tests, and therefore, less precise results. In the case of early plasma I-TAC, the 95% CI for the critical group is estimated as 727.53 - 2499.53 pg/ml, whereas that for non-critical is 459.35 - 799.35 pg/ml. No seemingly clear-cut difference between the two groups is obtained, even though a statistically significant difference is present, suggesting that the magnitude of the expected effect size is big, but a larger confirmatory study is needed to reach more meaningful conclusions [30]. Second, the variation among individual I-TAC values in each group was large, which may probably be due to the variation in sampling time (**Fig. 3E**). Third, we cannot rule out possible covariates and confounders that impact the early plasma I-TAC level. These factors may include some concomitant comorbidities, concomitant treatments such as the immediate supplemental oxygen and ventilation, or genetic variation [31]. Our analysis of hypertension, diabetes and hypothyroidism, the three most common comorbidities in this study, showed no association with changes in I-TAC levels.

In sum, our study provides first evidence that I-TAC levels may have prognostic value for COVID-19 disease severity, paving the way for in-depth follow-up studies that take important aspects such as comparable sampling time points into account.

## MATERIALS AND METHODS

### Samples

This study was approved by the ethical committee at the University of Oklahoma Health Sciences Center. The informed consent was obtained from all the patients enrolled in the COVID-19 biorepository at the University of Oklahoma IRB #11911 and three SARS-CoV-2 negative control donors. The patients were stratified as at mild, moderate, severe or critical stage based on the level of respiratory support required and need for admission (no O_2_ requirement, O_2_ requirement up to 6 L, high flow or bipap required, ventilation required) according to the NIH Coronavirus Disease 2019 (COVID-19) Treatment Guidelines [4]. Only moderate, severe or critical patients were hospitalized. The vast majority of patients had been admitted at the end of the week or beginning of the second week after symptom onset. Diagnosis was confirmed by nasopharyngeal PCR assay at the day or the next day of admission. The final stage of the disease each patient developed was determined during the course. Most of the baseline plasma samples were drawn within three days of hospitalization and before administration of any immunomodulatory agents. These samples represented nine moderate, 22 severe and five critical diseases. Plasma from these patients were collected multiple times during hospitalization from April 17, 2020 to December 18, 2020. The samples were stored at -80°C. Clinical characteristics were collected. Status at discharge were recorded.

### Antibody Array Assay

Human XL Cytokine Array Kits (ARY022B, R&D Systems, Minneapolis, MN) were used for cytokine screening following the manufacturer's instruction. Briefly, 20 μl plasma from each patient were diluted with 1.5 ml Array Buffer 6 and applied to one Cytokine Antibody Array Membrane, and incubated at 4°C for 16 h, followed by incubation with biotinylated detection antibody cocktail, Streptavidin-Horseradish Peroxidase (HRP) and chemiluminescent detection reagents. Signal was produced at the capture spot corresponding to the protein concentration in the plasma. Films were developed for different exposure times. Overexposed images exhibited by obvious background were not subjected to further analysis. Blot images were scanned with Color LaserJet Pro MFO M477fdn and quantified with Quick Spot image analysis (Western Vision Software, Salt Lake City, UT). Values used were from the images that generated values from most spots. Positive references on the membrane, spots coated with the same amount of biotinylated protein, were used as normalization controls among different batches of analysis.

### ELISA

Levels of candidate proteins/cytokines in plasma samples were determined using ELISA Kits for Gasdermin B (ELI-27962h, Nova Lifetech, Hongkong, China), IP10 (BMS284INST, ThermoFisher) and ITAC (EHCXCL11, ThermoFisher) following the manufacturer's instruction. Briefly, 50, 10 or 25 μl plasma were diluted with buffers provided with the kits and added to the microwells coated with specific capture antibodies. Corresponding standards were added alongside. Bound proteins were then detected by biotin-conjugated antibodies and streptavidin-HRP, followed by a substrate-based color reaction which reflects proportionally the amount of protein bound. The absorbance was read at 450 nm. Standard curve was made and concentration of specific protein/cytokine was calculated accordingly. Experiments were performed in duplicate and repeated three times.

### Statistics

The demographical variables and the protein abundance collected at admission were summarized with mean, standard deviation (SD), median, quartiles, and proportion. Both of the raw abundance and log2 transformed abundance were analyzed. The overall differences of these variables in four disease classes were tested by ANOVA test. For non-normally distributed continuous data, Kruskal-Wallis rank sum test was used. Jonckheere-Terpstra test was used for the test of increasing/decreasing trend of protein abundance among disease classes. We generated boxplots to explore the distribution of protein abundance among disease classes. The association between I-TAC and hospitalization time were assessed using Pearson's correlation test and linear regression. The pairwise comparisons of protein abundance between groups were conducted by the Wilcoxon rank sum test. For all the statistical tests, the significance level was 0.05.

## AUTHOR CONTRIBUTION

PM, YZ, RB and JHC designed study. YZ performed experiments. NIAH and JHC provided samples and clinical data. CX, YZ, PM and RB analyzed data. YZ, PM, JHC, CX and NIAH wrote paper that was edited and approved by all authors.

## SUPPLEMENTAL MATERIAL

Click here for supplemental data file.

All supplemental data for this article are available online at https://www.cell-stress.com/researcharticles/2021a-zhang-cell-stress/.

## Abbreviations:

COVID-19: – coronavirus disease 2019,
SARS-CoV-2: – severe acute respiratory syndrome coronavirus 2
