# Supplementary material for: Evaluation of I-TAC as a potential early plasma marker to differentiate between critical and non-critical COVID-19
Source: Cell Stress. 2021 Dec 21;6(1):6–16. doi: 10.15698/cst2022.01.262 (PMC8728569; doi:10.15698/cst2022.01.262)
Supplement: Supplementary file 1 [file ces-06-006-s01.pdf]

Supporting Information for

Evaluation of I-TAC as a potential early plasma marker to differentiate between critical and non-critical COVID-19

Yushan Zhang[1], Chao Xu[2], Nelson I. Agudelo Higuita[3], Resham Bhattacharya[4,5*], Jennifer Holter Chakrabarty [3*], Priyabrata Mukherjee[1,5*]

*Corresponding author. Email: Resham-Bhattacharya@ouhsc.edu
Jholter2@ouhsc.edu
Priyabrata-Mukherjee@ouhsc.edu

The supplementary materials include:

Fig. S1

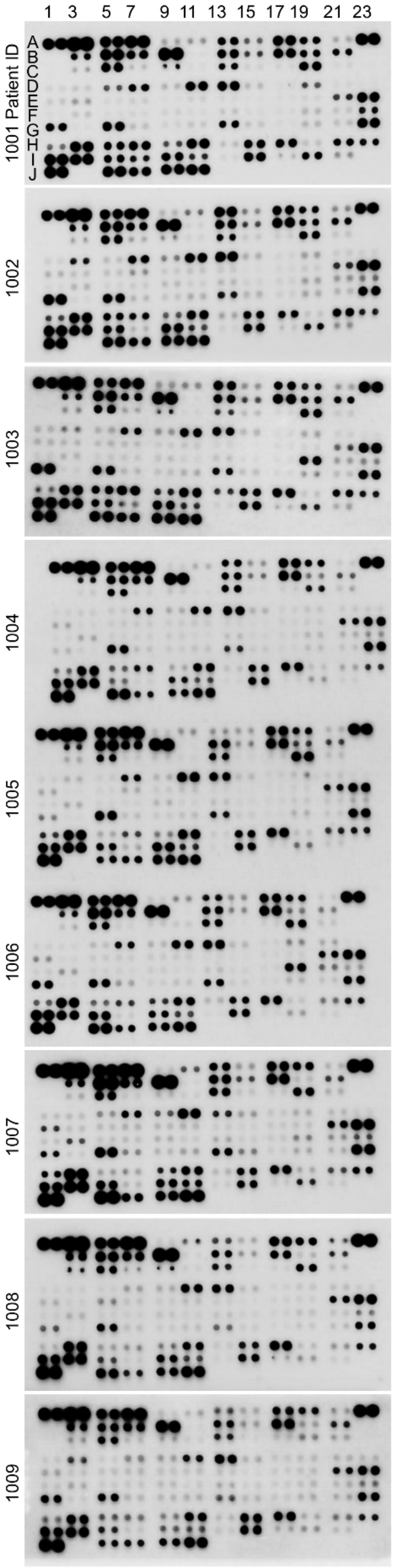

SPOTS
A1 (Reference Spots)
A2 (Reference Spots)
A3 (Adiponectin)
A4 (Adiponectin)
A5 (Apolipoprotein A-I)
A6 (Apolipoprotein A-I)
A7 (Angiogenin)
A8 (Angiogenin)
A9 (Angiopoietin-1)
A10 (Angiopoietin-1)
A11 (Angiopoietin-2)
A12 (Angiopoietin-2)
A13 (BAFF)
A14 (BAFF)
A15 (BDNF)
A16 (BDNF)
A17 (Complement Component C5/C5a)
A18 (Complement Component C5/C5a)
A19 (CD14)
A20 (CD14)
A21 (CD30)
A22 (CD30)
A23 (Reference Spots)
A24 (Reference Spots)
B3 (CD40 ligand)
B4 (CD40 ligand)
B5 (Chitinase 3-like 1)
B6 (Chitinase 3-like 1)
B7 (Complement Factor D)
B8 (Complement Factor D)
B9 (C-Reactive Protein)
B10 (C-Reactive Protein)
B11 (Cripto-1)
B12 (Cripto-1)
B13 (Cystatin C)
B14 (Cystatin C)
B15 (Dkk-1)
B16 (Dkk-1)
B17 (DPPIV)
B18 (DPPIV)
B19 (EGF)
B20 (EGF)
B21 (EMMPRIN)
B22 (EMMPRIN)
C3 (ENA-78)
C4 (ENA-78)
C5 (Endoglin)
C6 (Endoglin)
C7 (Fas Ligand)
C8 (Fas Ligand)
C9 (FGF basic)
C10 (FGF basic)
C11 (FGF-7)
C12 (FGF-7)
C13 (FGF-19)
C14 (FGF-19)
C15 (Flt-3 Ligand)
C16 (Flt-3 Ligand)
C17 (G-CSF)
C18 (G-CSF)
C19 (GDF-15)
C20 (GDF-15)
C21 (GM-CSF)
C22 (GM-CSF)
D1 (GRO-alpha)
D2 (GRO-alpha)
D3 (Growth Hormone)
D4 (Growth Hormone)
D5 (HGF)
D6 (HGF)
D7 (ICAM-1)
D8 (ICAM-1)
D9 (IFN-gamma)
D10 (IFN-gamma)
D11 (IGFBP-2)
D12 (IGFBP-2)
D13 (IGFBP-3)
D14 (IGFBP-3)
D15 (IL-1alpha)
D16 (IL-1alpha)
D17 (IL-1beta)
D18 (IL-1beta)
D19 (IL-1ra)
D20 (IL-1ra)
D21 (IL-2)
D22 (IL-2)
D23 (IL-3)
D24 (IL-3)
E1 (IL-4)
E2 (IL-4)
E3 (IL-5)
E4 (IL-5)
E5 (IL-6)
E6 (IL-6)
E7 (IL-8)
E8 (IL-8)
E9 (IL-10)
E10 (IL-10)
E11 (IL-11)
E12 (IL-11)
E13 (IL-12 p70)
E14 (IL-12 p70)
E15 (IL-13)
E16 (IL-13)
E17 (IL-15)
E18 (IL-15)
E19 (IL-16)
E20 (IL-16)
E21 (IL-17A)
E22 (IL-17A)
E23 (IL-18 BPa)
E24 (IL-18 BPa)
F1 (IL-19)
F2 (IL-19)
F3 (IL-22)
F4 (IL-22)
F5 (IL-23)
F6 (IL-23)
F7 (IL-24)
F8 (IL-24)
F9 (IL-27)
F10 (IL-27)
F11 (IL-31)
F12 (IL-31)
F13 (IL-32)
F14 (IL-32)
F15 (IL-33)
F16 (IL-33)
F17 (IL-34)
F18 (IL-34)
F19 (IP-10)
F20 (IP-10)
F21 (I-TAC)
F22 (I-TAC)
F23 (Kallikrein 3)
F24 (Kallikrein 3)
G1 (Leptin)
G2 (Leptin)
G3 (LIF)
G4 (LIF)
G5 (Lipocalin-2)
G6 (Lipocalin-2)
G7 (MCP-1)
G8 (MCP-1)
G9 (MCP-3)
G10 (MCP-3)
G11 (M-CSF)
G12 (M-CSF)
G13 (MIF)
G14 (MIF)
G15 (MIG)
G16 (MIG)
G17 (MIP-1alpha/MIP-1beta)
G18 (MIP-1alpha/MIP-1beta)
G19 (MIP-3alpha)
G20 (MIP-3alpha)
G21 (MIP-3beta)
G22 (MIP-3beta)
G23 (MMP-9)
G24 (MMP-9)
H1 (Myeloperoxidase)
H2 (Myeloperoxidase)
H3 (Osteopontin)
H4 (Osteopontin)
H5 (PDGF-AA)
H6 (PDGF-AA)
H7 (PDGF-AB/BB)
H8 (PDGF-AB/BB)
H9 (Pentraxin-3)
H10 (Pentraxin-3)
H11 (PF4)
H12 (PF4)
H13 (RAGE)
H14 (RAGE)
H15 (RANTES)
H16 (RANTES)
H17 (RBP-4)
H18 (RBP-4)
H19 (Relaxin-2)
H20 (Relaxin-2)
H21 (Resistin)
H22 (Resistin)
H23 (SDF-1alpha)
H24 (SDF-1alpha)
I1 (Serpin E1)
I2 (Serpin E1)
I3 (SHBG)
I4 (SHBG)
I5 (ST2)
I6 (ST2)
I7 (TARC)
I8 (TARC)
I9 (TFF3)
I10 (TFF3)
I11 (TfR)
I12 (TfR)
I13 (TGF-alpha)
I14 (TGF-alpha)
I15 (Thrombospondin-1)
I16 (Thrombospondin-1)
I17 (TNF-alpha)
I18 (TNF-alpha)
I19 (uPAR)
I20 (uPAR)
I21 (VEGF)
I22 (VEGF)
J1 (Reference Spots)
J2 (Reference Spots)
J5 (Vitamin D BP)
J6 (Vitamin D BP)
J7 (CD31)
J8 (CD31)
J9 (TIM-3)
J10 (TIM-3)
J11 (VCAM-1)
J12 (VCAM-1)
J23 (NC)
J24 (NC)

Figure S1. Typical images of cytokine array. Plasmas (20 µl) from each patient or control donor was diluted with 1.5 ml array buffer and applied to one cytokine antibody array membrane. Films were developed for different duration from 3 min to 16 hrs. Patient or donor ID numbers are labeled on the left of each image. Cytokine name and location in the membrane are shown in the right table. (To be continued)

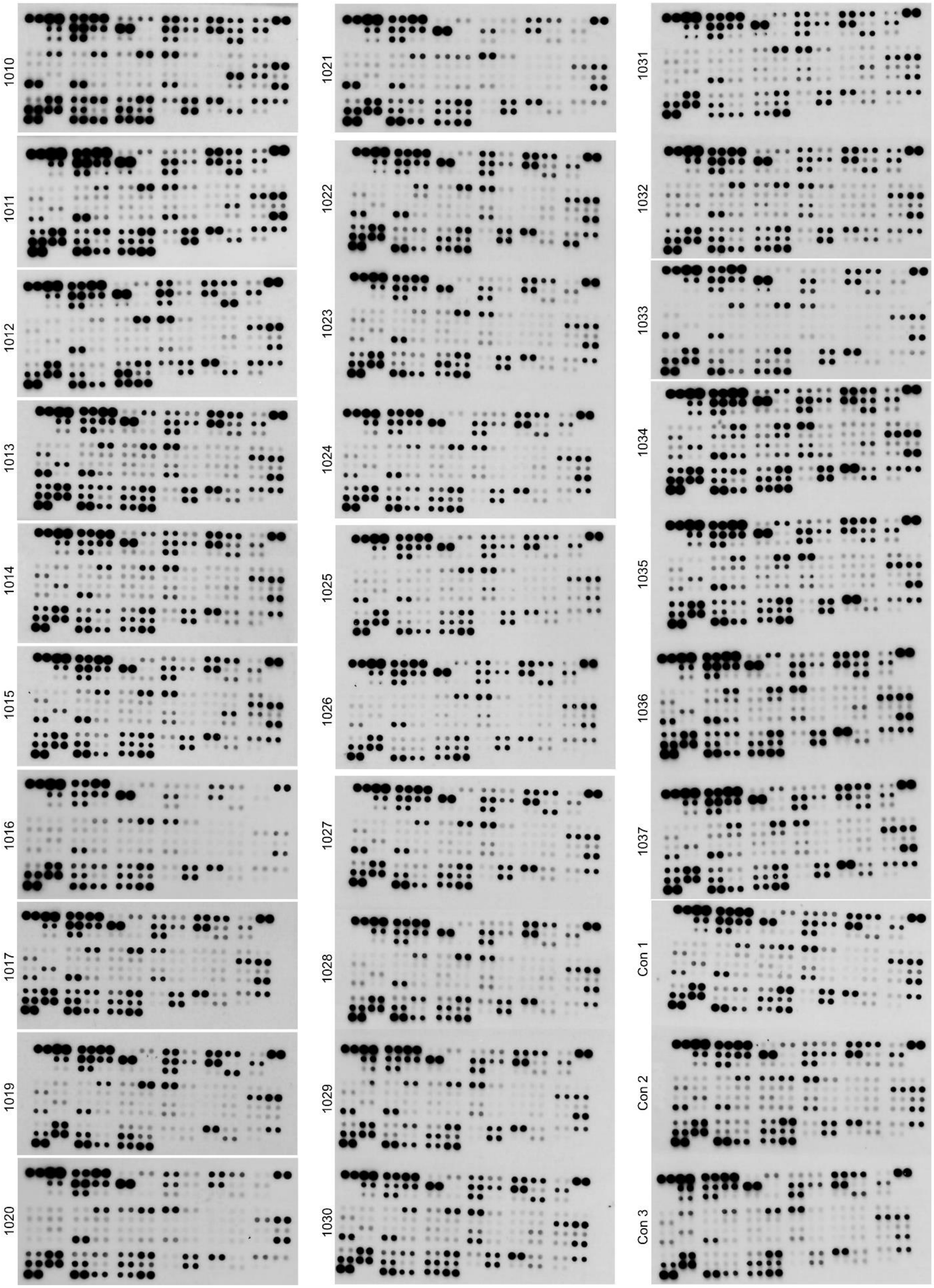

Figure S1. Continued
